# COX-2 selective inhibition reverses the trophic properties of gastrin in colorectal cancer

**DOI:** 10.1038/sj.bjc.6600495

**Published:** 2002-08-27

**Authors:** M Yao, D H Song, B Rana, M M Wolfe

**Affiliations:** Section of Gastroenterology, Boston University School of Medicine and Boston Medical Center, 605 Albany Street, Room 504, Boston, Massachusetts, MA 02118, USA

**Keywords:** gastrin, cyclo-oxygenase, colorectal cancer, trophic properities

## Abstract

Gastrin is a gastrointestinal peptide that possesses potent trophic properties on both normal and neoplastic cells of gastrointestinal origin. Previous studies have indicated that chronic hypergastrinaemia increases the risk of colorectal cancer and cancer growth and that interruption of the effects of gastrin could be a potential target in the treatment of colorectal cancer. Here we demonstrate that gastrin leads to a dose-dependent increase in colon cancer cell proliferation and tumour growth *in vitro* and *in vivo*, and that this increment is progressively reversed by pretreatment with the cyclo-oxygenase-2 inhibitor NS-398. Gastrin was able to induce cyclo-oxygenase-2 protein expression, as well as the synthesis of prostaglandin E_2_, the major product of cyclo-oxygenase. Moreover, gastrin leads to approximately a two-fold induction of cyclo-oxygenase-2 promoter activity in transiently transfected cells. The results of these studies demonstrate that cyclo-oxygenase-2 appears to represent one of the downstream targets of gastrin and that selective cyclo-oxygenase-2 inhibition is capable of reversing the trophic properties of gastrin and presumably might prevent the growth of colorectal cancer induced by hypergastrinaemia.

*British Journal of Cancer* (2002) **87**, 574–579. doi:10.1038/sj.bjc.6600495 www.bjcancer.com

© 2002 Cancer Research UK

## 

The polypeptide hormone gastrin was identified nearly 100 years ago, and its role in the physiology of gastric acid secretion is well-established (Modlin *et al*, 1997). Another biological property attributed to gastrin is its trophic effect on gastrointestinal (GI) mucosa, including its role in the pathogenesis of GI carcinogenesis (Koh *et al*, 1999; Stepan *et al*, 1999; Koh and Chen, 2000). Previous epidemiological studies have indicated that chronic hypergastrinaemia constitutes a risk factor for the development of colorectal cancer (Hakanson *et al*, 1986; Sundler *et al*, 1986; Wolfe, 1992; Lamberts *et al*, 1999; Singh *et al*, 2000; Watson and Smith, 2001). Significant hypergastrinaemia occurs in association with a number of clinical conditions, including pernicious anaemia and Zollinger-Ellison syndrome and following the development of potent acid suppression in response to the administration of proton pump inhibitors (Klingensmith *et al*, 1999). The increased incidence of colorectal cancer in hypergastrinaemic patients appears to occur as a result of an increased rate of proliferation of normal colonic epithelium, thus increasing the chance of sporadic mutations. Hypergastrinaemia may also enhance the proliferation and progression of colorectal adenomas (Thorburn *et al*, 1998; Watson and Smith, 2001).

Epidemiological studies have demonstrated a 40–50% reduction in mortality from colorectal cancer in individuals taking nonsteroidal anti-inflammatory drugs (NSAIDs), which appears to reduce the risk of colorectal cancer (CRC) by inhibiting cyclo-oxygenase (COX), a key enzyme involved in the metabolic conversion of arachidonic acid to prostaglandins (Giovannucci *et al*, 1994; Giardiello *et al*, 1995). Numerous studies have shown that the expression of COX-2, one of the two isoforms of COX, is increased significantly in colonic neoplasms compared with normal colonic mucosa, and that COX-2 plays an integral role in colon cancer tumorigenesis and proliferation (Sheng *et al*, 1997; Barnes *et al*, 1998; Barnes and Lee, 1998; Sawaoka *et al*, 1998; Tsujii *et al*, 1998). However, the cellular and molecular mechanisms governing any possible relationship between gastrin and COX during GI tumour growth have not been elucidated. The purpose of this study was to examine whether COX-2 inhibition is able to reverse the trophic properities of gastrin in CRC.

## MATERIALS AND METHODS

### Cell culture

MC-26 cells, a transplantable mouse colon cancer cell line that possesses both COX-2 and functional gastrin receptors (Singh *et al*, 1986), were obtained from Dr KK Tanabe (Boston, MA, USA). MC-26 cells were maintained in Dulbecco's Modified Eagle Media (DMEM; Life Technologies, Inc, Gaithersburg, MD, USA) supplemented with 10% fetal calf serum and antibiotics (100 U ml^−1^ penicillin and 100 μg ml^−1^ streptomycin) at 37°C in a humidified atmosphere of 95% air per 5% CO_2_.

### Proliferation studies

DNA synthesis was estimated by the measurement of [^3^H]thymidine incorporation into cellular DNA. Cells (100 000 ml^−1^) were seeded onto 12- or 24-well plates and allowed to attach overnight, after which they were incubated in serum-free medium for another 24 h. This incubation was followed by treatment with different concentrations of gastrin-17 (G-17; Peninsula Laboratories, San Carlos, CA, USA) in the presence or absence of the specific COX-2 inhibitor, NS-398 (Cayman Chemical, Ann Arbor, MI, USA). NS-398 and gastrin-17 were dissolved in DMSO and 30 mM NH_4_HCO_3_, respectively, as stock solutions. One μCi ml^−1^ of [^3^H]thymidine (New England Nuclear Products, Boston, MA, USA) was added and allowed to incorporate for 6 h at 37°C. Cells were washed with cold phosphate buffered saline (PBS) three times. Cold 10% trichloracetate (TCA) was added to cells and maintained at 4°C 30 min, after which cells were washed again with cold PBS three times and lysed in 0.1 N NaOH per 10% SDS. Radioactivity was counted in a liquid scintillation counter, and data were expressed as percentage of control±standard error (s.e.) of several experiments.

### Mouse colorectal cancer model

Six- to ten-week old male BALB/C mice were obtained from Taconic (Germantown, NY, USA). MC-26 cells were harvested from subconflent cultures using trypsin-EDTA, followed by centrifugation at 300 G for 15 min at room temperature. Cells were then resuspended in serum-free DMEM or Hank's Balanced Salt Solution (Life Technologies, Inc, Gaithersburg, MD, USA), and the cell number was adjusted to a final concentration of 100 000 cells per ml. Using a 27-gauge needle and a 1 ml syringe, 100 μl of tumour cell suspension was injected subcutaneously into the flanks of mice. All animal studies were conducted using a protocol approved by the Animal Care and Use Committee at Boston University Medical Center and in accordance with UKCCCR Guidelines (Workman *et al*, 1998).

### Animal study procedure

NS-398, dissolved in DMSO, was administered by oral gavage once daily. G-17 was dissolved in 0.9% NaCl and was administrated using an implanted Alzet® osmotic pump (Alza corporation, Palo Alto, CA, USA). For osmotic pump insertion, animals were anaesthetised using intraperitoneal pentobarbital (65 mg kg^−1^) injection. An incision ∼0.8 cm in length was made, and the osmotic pump was implanted subcutaneously. After tumour cell injection, mice were randomly divided into four groups (10 animals per group) on day 0, followed by treatment with different test reagents:

Group 1 (control group): Infusion of 0.9% NaCl by osmotic pump and DMSO (vehicle) 0.1 ml by gavage;

Group 2: Infusion of G-17 10 nmol kg^−1^ h^−1^ by osmotic pump and DMSO 0.1 ml by gavage;

Group 3: Infusion of G-17 10 nmol kg^−1^ h^−1^ by osmotic pump along with NS-398 1 mg kg^−1^ body weight by gavage; and

Group 4: Infusion of G-17 10 nmol kg^−1^ h^−1^ by osmotic pump along with NS-398 10 mg kg^−1^ body weight by gavage.

Subcutaneous tumour size was determined after day 7 by measuring the longest and shortest diameters of the tumour at 2–3 day intervals. Tumour volume (mm^3^) was calculated using the standard formula: tumour volume=(shortest diameter)^2^×(longest diameter)×0.5. After sacrificing the mice on day 18, tumours were excised and weighed and measured. Tumour tissue was flash frozen in liquid nitrogen and stored at –70°C, and a portion of the tissue was fixed with 10% formalin for histological examination.

### Prostaglandin E_2_ assay

MC-26 cells (100 000 ml^−1^) were seeded onto six-well plates and allowed to attach overnight. Cells were then cultured in serum-free medium for another 24 h, followed by treatment with 20 nM of G-17. To evaluate the activity of COX, prostaglandin E_2_ (PGE_2_), the major metabolite of arachidonic acid metabolism, was measured by an enzyme immunoassay (EIA) kit (Cayman Chemical, Ann Arbor, MI, USA) in culture media maintained at 20°C using the protocol provided by manufacturer. Measurements were made in triplicate in three separate experiments.

### Transfection and reporter gene assays

To examine transcriptional regulation of the COX-2 promoter by gastrin, MC-26 cells were transiently transfected with 742-kb COX-2 promotor (kindly provided by Dr H Herschman, UCLA) or control plasmid pGL3-Luc (Promega, Madsion, WI, USA) in the presence of G-17 using Lipofectamine™ Reagent (Life Technologies, Inc, Gaithersburg, MD, USA). A β-galactosidase-expressing plasimd was included in each transfection to monitor the transfection efficiency. For luciferase assay, transfected cells were washed twice with phosphate buffered saline (PBS, pH 7.4) and lysed in 200 μl of lysis buffer following the manufacturer's instructions (BD PharMingen, San Diego, CA, USA). β-galactosidase activity in 50 μl of the cell lysate was determined after 5–20 min incubation at 37°C with 2 mM chlorophenol red β-galactopyranoside (Boehringer Mannheim, Indianapolis, IN, USA) in 20 mM MgCl_2_, 0.1 mM MnCl_2_, 45 mM 2-mercaptoethanol, and 100 mM NaHPO_4_, pH 8.0. The reaction was stopped by adding 500 μl of 0.5 M EDTA, pH 8.0, and the absorbency at 570 nm measured using a spectrophotometer. With each experiment, luciferase activity was determined in duplicate and normalized to β-galactosidase activity for each dish.

### Western blot hybridization

Mouse COX-2 and cyclin D1 monoclonal antibodies were purchased from BD transduction laboratories (Lexington, KY, USA). To extract protein from cells, MC-26 cells cultured under different conditions were harvested and lysed in RIPA buffer (PBS, 1% NP-40, 0.5% sodium deoxycholate, 0.1% SDS, 100 ng ml^−1^ PMSF, 66 ng ml^−1^ aprotinin). To extract protein from tissues, 0.1 g of tumour tissue was placed in 2.0 ml of cold RIPA buffer and homogenized for 1 min with Polytron-Aggregate (Kinmatica, Luzern, Switzerland). After removal of cell debris by centrifugation, total protein of cells or tissues was determined by BCA protein assay (Pierce chemical, Rockford, IL, USA). Protein was mixed with gel loading buffer (50 mM Tris pH 6.8, 2% SDS, 10% glycerol, 2% 2-mercaptoethanol, 0.1% bromphenol blue) and heated for 10 min at 100°C. Samples containing 20–40 μg protein were loaded on a 10–12% SDS–PAGE gel and then electrophoretically transferred to a polyvinylidene difluoride membrane in transfer buffer (25 mM Tris, 190 mM glycine, 20% methanol). The blots were blocked with 7% dry milk for 1 h at room temperature and incubated with the primary antibody overnight. They were then washed three times for 15 min each in Tris-buffered saline, containing 0.05% Tween-20. The blots were further incubated with the anti-mouse IgG antibody (Sigma) for 1 h at room temperature. After washing three times, blots were incubated with luminous ECL reagent (Pierce chemical, Rockford, IL, USA) for 10 s to 2 min and exposed to Kodak X-ray film. Protein bands were quantified by laser densitometry.

### Immunohistochemistry

Proliferating cell nuclear antigen (PCNA) monoclonal antibodies were purchased from BD transduction laboratories (Lexington, KY, USA). Paraffin-embedded specimens were deparaffinized and incubated with PCNA antibody for 2 h at 37°C. The specimens were then incubated with the secondary antibody, anti-mouse IgG, for 1 h at 37°C and stained by the avidin-biotin peroxidase complex (ABC) method using the ABC staining system (Santa Cruz Biotech, Santa Cruz, CA, USA). They were visualized by 3,3-diaminobenzidine (DAB) staining and counterstaining with haematoxylin. To confirm the specificity of the mouse PCNA antibody, tonsil specimens was used as a positive control. The PCNA index was evaluated by counting the number of PCNA-positive staining cells out of a total of 500 tumour cells: PCNA index= (number of PCNA-positive-staining cells per 500 cells counted)×100%.

### Statistical analysis

One-way ANOVA was performed to compare [^3^H]thymidine incorporation, tumour volume and weight, PCNA index, and densitometric values of Western blot bands among the different animal groups, followed by Tukey's procedure for paired comparison. The two-tailed *t*-test was used to compare PGE_2_ levels in the different conditions. Statistical significance was assigned if *P*<0.05.

## RESULTS

### Cell proliferation

**Figure 1:**
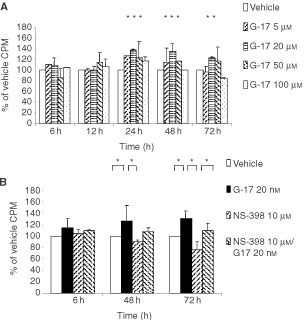
[^3^H]thymidine incorporation of colon cancer cells (MC-26) treated with gastrin-17 (G-17) in the absence or presence of the COX-2 selective inhibitor NS-398. DNA synthesis was estimated by [^3^H]thymidine incorporation into cellular DNA, as described in the Materials and Methods section, under various conditions: gastrin-17 (**A**) and the combination of G-17 and NS-398 (**B**). *P<0.05.

The addition of G-17 led to an increase in [^3^H]thymidine incorporation, with ∼40% increase detected using at a G-17 concentration of 20 nM. NS-398, a COX-2 selective inhibitor, decreased [^3^H]thymidine incorporation in a dose-dependent manner. When MC-26 cells were incubated in culture media containing both 20 nM G-17 and 10 μM NS-398, [^3^H]thymidine incorporation was 110.0% of control (Figure 1

), indicating that the enhancement in cell proliferation induced by gastrin could be partially attenuated by COX-2 inhibition with NS-398.

### Tumour growth

**Figure 2:**
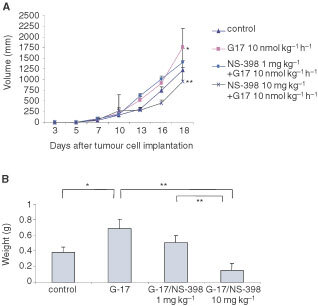
Effect of gastrin-17 (G-17) or/and the COX-2 selective inhibitor (NS-398) on colon cancer growth *in vivo*. MC-26 cells were injected subcutaneously in the flank of 6 to 10-week old male BALB/c mice. Subcutaneous tumour size was determined from day 7 by measuring the longest and shortest diameter of the tumour at 2–3 days intervals. (**A**) Tumour volume (mm^3^) was calculated by a standard formula: Volume=(the shortest diameter)^2^ ×(the longest diameter)×0.5. (**B**) Tumour weight was measured on day 18 after tumour was removed from sacrificed mice. *P<0.05. **P<0.01.

In animal studies, 6- to 10-week old BALB/C mice were inoculated with MC-26 cells subcutaneously on day 0. Figure 2

depicts the effects of G-17 and NS-398 on tumour volume and tumour weight at the end of the period of observation (day 18). Twenty per cent of G-17 treated mice died as a result of heavy tumour burden. Both tumour volume and weight in mice treated with G-17 (10 nmol kg^−1^ h^−1^) were significantly greater than those in the control group (tumour volume 1761.8±427.6 mm^3^ *vs* 1220.2±224.0 mm^3^, tumour weight 0.53±0.04 g *vs* 0.38±0.07 g, *P*<0.05). In contrast, no significant differences in tumour volume and weight from control was detected in mice treated with both NS-398 (1 mg kg^−1^) and G-17 (10 nmol kg^−1^ h^−1^). Moreover, tumour growth stimulated by G-17 was reversed by both low- (1 mg kg^−1^) and high-dose (10 mg kg^−1^) NS-398. The latter not only suppressed gastrin-induced tumour growth, but also unstimulated (control) tumour growth (tumour volume: 955.8±325.1 mm^3^ *vs* 1220.2±224.0 mm^3^, *P*<0.05; tumour weight: 0.15±0.09 g *vs* 0.38±0.07 g, *P*<0.01).

**Figure 3:**
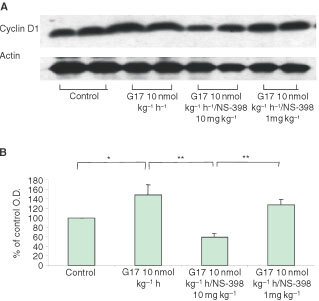
Western blot of cyclin D1 expression. (**A**) in MC-26 colon cancer tissues treated with gastrin-17 (10 nmol kg^−1^ h^−1^) in the absence or presence of NS-398 (1 and 10 mg kg^−1^). (**B**) Analysis of Western blot densitometry. *P<0.05, **P<0.01.

Consistent with tumour growth, cyclin D1 protein and PCNA index in tumour tissues were significantly greater in the tumour tissue of G-17 treated mice. Low-dose NS-398 (1 mg kg^−1^) partially attenuated gastrin-induced cyclin D1 and PCNA, while high-dose NS-398 (10 mg kg^−1^) significantly decreased both of them to values significantly less than unstimulated conditions (Figures 3

and 4

**Figure 4:**
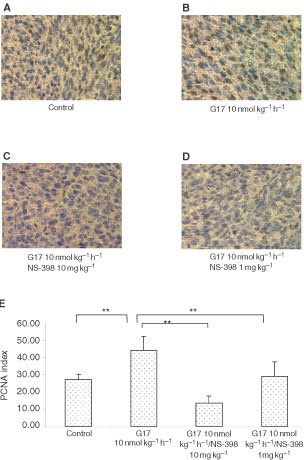
Proliferating cell nuclear antigen (PCNA) in colon cancer treated with gastrin-17 in the absence or presence of NS-398. Cells were injected subcutaneously in the flank of 6 to 10-week old male BALB/c mice. Mice were sacrificed on day 18, and PCNA index was determined by immunohistochemistry, as described in the Materials and Methods section (**A**: Control, **B**: Gastrin-17 10 nmol kg^−1^ h^−1^, **C**: Gastrin-17 10 nmol kg^−1^ h^−1^ and NS-398 10 mg kg^−1^, **D**: Gastrin-17 10 nmol kg^−1^ h^−1^ and NS-398 1 mg kg^−1^, **E**: Quantification of PCNA index. *P<0.05, **P<0.01).

).

### COX-2 promoter activity, COX-2 protein expression and prostaglandin E_2_ (PGE_2_) levels

**Figure 5:**
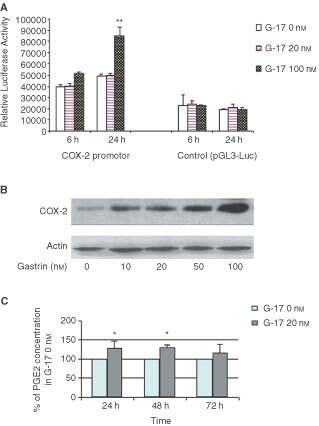
COX-2 promoter activity, COX-2 protein and prostaglandin synthesis induced by gastrin-17. (**A**) COX-2 promoter activity, (**B**) protein expression, and (**C**) change of prostaglandin levels in MC-26 cells in response to gastrin. *P<0.05, **P<0.01.

To study whether G-17 is capable of inducing COX-2 transcription, cells were transiently transfected with a COX-2 promoter luciferase construct, and luciferase assays were performed, as described above. COX-2 promoter activity was induced approximately two-fold in transfected cells incubated in presence of G-17 (100 nM) at 24 h. In addition, COX-2 protein expression was significantly increased in MC-26 cells incubated in the presence 10, 20, 50 and 100 nM G-17 at 24 h. When cells were incubated with 20 nM G-17, levels of PGE_2_, the major product of cyclo-oxygenase, were significantly increased at 24 h (215.9±13.6 pg well^−1^ *vs* 170.8±27.9 pg well^−1^, *P*<0.05) and 48 h (350.8±39.7 pg well^−1^ *vs* 272.2±35.6 pg well^−1^, *P*<0.05) (Figure 5

).

## DISCUSSION

Previous studies have reported that gastrin and NSAIDs possess opposing effects on cell proliferation. Gastrin has long been recognized as a mitogenic factor that stimulates the growth of pre-existing tumours of GI origin (Baldwin and Shulkes, 1998; Smith and Watson, 2000; Dockray *et al*, 2001). Interruption of the effects of gastrin as a potential target in the treatment of colorectal cancer, using several different approaches, such as the gastrin (CCK-B or CCK-2) receptor antagonists, proglumide and benotript, has been assessed (Watson *et al*, 1992a,b). The major drawback of these compounds is their lack of potency, with relatively high concentrations required to displace amidated G-17_._ L-365,260 has a greater affinity for the gastrin receptor than proglumide and has been shown to reverse gastrin-stimulated growth of GI tumour cell, both *in vitro* and *in vivo (*Piontek and Hengels, 1993; Watson *et al*, 1991*)*. However, this antagonist does not appear to inhibit basal growth of the tumour and lacks the capacity to interact with alternate gastrin receptor subtypes. In contrast, the effectiveness of anti-gastrin antibodies in inhibiting tumour growth has been demonstrated in several animal models of colorectal cancer (Watson *et al*, 1995, 1996, 2000).

The benefit of nonselective COX inhibitors in preventing tumorigenesis and tumour growth has been demonstrated in numerous studies (Dubois and Smalley, 1996; Levy, 1997). However, the use of these agents is often associated with the development of serious adverse GI events. COX-2 selective inhibitors are thought to exert similar anti-inflammatory and antimitogenic effects, but with diminished toxicity (Wolfe *et al*, 1999). N-[2-(cyclohexyloxy)-4-nitrophenyl]-methanesulphonamide (NS-398) is a sulfonamide derivative that inhibits COX-2 specifically with an IC_50_ of 30 nM. It does not affect COX-1 enzyme activity at concentrations exceeding 100 μM, and it inhibits COX-1 dependent prostanoid production only minimally even at doses >200 mg kg^−1^ (Futaki *et al*, 1994; Gierse *et al*, 1995). The results of these studies suggest the possibility of a functional relationship between gastrin and COX-2 expression and demonstrate that COX-2 selective inhibition is capable of reversing the trophic properties of growth on colorectal adenocarcinoma. Because COX-2 selective inhibition has been shown to possess antineoplastic properties with few adverse GI events, the use of these agents may potentially represent a novel therapeutic approach to reduce the risk of colon cancer associated with hypergastrinaemia.

Despite these observations, the cellular and molecular mechanisms governing any potential relationship between COX-2 and gastrin require further clarification. In this study, when MC-26 colorectal cancer cells were incubated in the presence of gastrin, significant increases in COX-2 protein levels and COX-2 promoter activity was detected, compared with control conditions. Furthermore, using a sensitive enzyme immonoassay (EIA) for the measurement of PGE_2_, modest, but significant, increases in PGE_2_ levels in response to 20 nM gastrin were observed. While further studies are necessary to clarify any possible functional relationship, the present results do imply that a COX-2 mediated pathway may be stimulated by gastrin and may contribute to its trophic effects on colorectal cancer.

Cyclin D1 is a protein involved in cell cycle regulation in both normal and neoplastic cells (Hunter and Pines, 1994). In the G1 (resting) phase of the cell cycle, cyclin D1 along with its cyclin dependent kinase (CDK) partner, is responsible for transition to the S (DNA synthesis) phase (Sherr, 1996). Overexpression of cyclin D1 releases a cell from its normal control and causes transformation to a malignant phenotype. Previous studies have demonstrated that cyclin D1 is increased in adenomatous polyps and in both sporadic and familial forms of colorectal cancer (Motokura and Arnold, 1993; Bartkova *et al*, 1994; Arber *et al*, 1996, 1997). Consistent with these prior observations, in the present study, gastrin increased cyclin D1 levels *in vivo* and *in vitro*, an effect that was reversed with NS-398. In addition to cyclin D1, PCNA functions as an auxiliary protein to DNA polymerase gamma and as a co-factor in DNA synthesis. The synthesis and expression of PCNA are enhanced in proliferating cells including those that are tumour-derived. Determination of PCNA represents one of the most reliable methods for evaluating proliferation in cells and tissues (Prosperi, 1997). In the present study, the PCNA index was significantly increased in gastrin-treated tumours when compared with control. Moreover, similar to studies assessing cyclin D1, the addition of NS-398 (10 mg kg^−1^ body weight) reversed the gastrin-induced increase in PCNA expression.

In conclusion, the results of these studies demonstrate that COX-2 might represent one of the downstream targets of gastrin and that selective COX-2 inhibition is capable of reversing the trophic properties of gastrin and presumably prevent growth of CRC induced by hypergastrinaemia. In addition to its effects on cyclin D1 and PCNA, it is certainly possible that other intracellular pathways are involved in mediating the trophic properties of gastrin on neoplastic proliferation. Although the therapeutic implications are obvious, further studies will be necessary to elucidate the cellular and molecular mechanisms governing any potential functional relationship between COX-2 and gastrin.
